# Recent Knowledge Regarding the Epidemiology, Exacerbating Factors, and Treatment of Rheumatoid Arthritis Complicated by Interstitial Lung Disease

**DOI:** 10.3390/jcm15135175

**Published:** 2026-07-02

**Authors:** Yoshiro Horai

**Affiliations:** Department of Rheumatology, Sasebo City General Hospital, Sasebo 857-8511, Japan; yoshiro.horai@hospital.sasebo.nagasaki.jp

**Keywords:** biological disease-modifying antirheumatic drugs, interstitial lung disease, Janus kinase inhibitors, methotrexate, rheumatoid arthritis

## Abstract

Rheumatoid arthritis (RA) is a systemic rheumatic disease. Its most prominent characteristic is synovitis, which manifests clinically as arthritis, resulting in joint damage. Interstitial lung disease (ILD) is an extraarticular manifestation of RA that hinders therapeutic goals, affects life prognosis, and can be worsened by the administration of disease-modifying antirheumatic drugs (DMARDs). Therefore, proper management of ILD, including consideration of risk factors such as the systemic disease activity of RA and autoantibodies, and early detection with high-resolution computed tomography are required at the introduction of DMARDs. Methotrexate, a class of DMARD used as the anchor drug for RA treatment, has been recognized as likely to worsen RA-ILD. However, there are currently reports suggesting that methotrexate may have beneficial effects on RA-ILD. Conversely, several studies have also shown exacerbations of ILD with the administration of biological DMARDs, which are thought of as tolerable for patients with RA-ILD. Rheumatologists should be wary of emergence or changes in the activity of ILD and arthritis during treatment of any kind with DMARDs. Further studies are warranted to clarify underlying ethnic factors, such as the possible effects of antimelanoma differentiation-associated gene 5 antibodies on RA-ILD.

## 1. Introduction

Rheumatoid arthritis (RA) is a systemic rheumatic disease (RD); its most prominent characteristic is synovitis, which manifests clinically as arthritis, resulting in joint pain, swelling, and damage [1]. Like those with arthritis, patients with RA can also present with complicating extraarticular manifestations, such as interstitial lung disease (ILD), vasculitis, and heart disease [2]. While the overall incidence of other extraarticular manifestations has decreased, that of ILD has not, which might be attributed to the prevalence of disease-modifying antirheumatic drugs (DMARDs) that are effective for arthritis but have the potential to exacerbate ILD [3]. ILD can develop at any stage of RA [4] and has been reported to hinder the achievement of therapeutic goals, i.e., it is a risk factor for difficult-to-treat RA [5]. It can also affect life prognosis, as is the case with other RDs such as idiopathic inflammatory myopathies and Sjögren’s disease [6].

It is recommended to introduce DMARDs soon after diagnosis of RA in order to obtain persistent low disease activity or remission [7]. However, DMARD-induced onset or exacerbation of ILD can cause life-threatening damage to the lungs in patients with RA. Methotrexate (MTX), a class of conventional synthetic DMARDs (csDMARDs), is recognized as the anchor drug for treatment for RA [8] and is known to cause drug-induced/worsening of ILD [9]. However, several studies have reported that MTX is not associated with worsening of ILD or may even have protective action against RA-ILD [10,11,12], suggesting that MTX might be beneficial even for patients with RA-complicating ILD depending on the degree of severity. These conflicting results are even mentioned in the latest practice guidelines by both the American College of Rheumatology (ACR)/American College of Chest Physicians (ACP) [6] and the European Respiratory Society (ERS)/European Alliance of Associations for Rheumatology (EULAR) [13]. Molecular targeted drugs, including biological DMARDs (bDMARDs) and Janus kinase inhibitors (JAKis), are considered for patients with RA refractory to MTX or other csDMARDs. Whereas molecular target therapy has revolutionized treatment for RA, both beneficial and detrimental results have been observed in RA-ILD, even within each class of molecular targeted drug.

Recently, the ACR/ACP [6] and the ERS/EULAR [13] each published guidelines for ILD associated with RDs, which describe the present state of general knowledge on recommended drugs, including DMARDs, for ILD associated with RDs, including RA. Advances in DMARD therapy developed by recent large clinical trials mainly conducted by countries belonging to the ACR or EULAR have brought innovation in treatment for RA all over the world. However, there are differences in the prevalence rates, clinical characteristics, and drug indication of RA or RA-ILD among countries, which requires attending physicians to make decisions for treatment of RA-ILD considering clinical situations in their own regions, as well as individual patient characteristics. Within EULAR countries, the French Society of Rheumatology has published recommendations for RA treatment including RA-ILD [14], and the Italian Society for Rheumatology has published guidelines for the treatment of RA-ILD [15]. Among Asian countries, the Japanese Respiratory Society (JRS)/Japan College of Rheumatology (JCR) jointly published a guide for the treatment of ILD associated with RDs, including RA [16]. However, considering the above-mentioned reports of inconsistent conclusions within one class of DMARD, the assessment of existence and severity of concomitant ILD in each patient and the choice of DMARDs based on ever-changing knowledge on RA-ILD would be important for attending physicians treating patients with RA.

In this review, we detail recent knowledge from around the world regarding RA coexisting with ILD and its treatment, aiming to contribute to better control of RA and coexisting ILD.

## 2. Epidemiology and Characteristics of ILD in Patients with RA

### 2.1. RA-ILD Epidemiology, Diagnosis, and Prognosis

While complication rates of ILD in patients with RA have previously been reported at around 2%, this increased to 4 to 15% in cases where high-resolution computed tomography (HRCT), pulmonary function test (PFT), or both were used for detection [10]. ILD was found in 11% of the study patients in a recent study conducted in hospitals in the United States on patients with RA of two or less years in duration, in which HRCT for ILD screening was included [17]. The complication rates of ILD in patients with RA vary depending on the study populations and methods, such as tools used for detecting ILD [18].

The detection of RA-ILD and assessment of its severity require a multidisciplinary approach. Lung biopsy can be considered for definitive diagnosis, as with ILD associated with other RDs, but clinical and radiographic diagnoses are performed in many cases due to the invasiveness of biopsy and high diagnostic accuracy in usual interstitial pneumonia (UIP) and idiopathic pulmonary fibrosis patterns on HRCT [19]; surgical lung biopsy is not recommended for the screening of RA-ILD in current guidelines [13,20]. UIP is the most common radiographic pattern on HRCT in RA-ILD, with nonspecific interstitial pneumonia (NSIP) being second, both of which are found in chronic forms. Other than UIP and NSIP, organizing pneumonia and diffuse alveolar damage can be found in acute or subacute forms of RA-ILD, which requires different therapies than chronic forms [16]. HRCT has enabled pulmonologists and rheumatologists to detect ILD at subclinical stages, i.e., before the development of physical disturbances or decreased pulmonary function [21].

It has been reported that even subclinical ILD can affect mortality in patients with RA. Zhang et al. analyzed patients with RA from the Mass General Brigham Biobank, including 151 patients with ILD, 70 patients with bronchiectasis, and 980 patients without lung complications. Patients with RA-ILD with UIP showed the worst mortality rate, which suggests the importance of early diagnosis for this group [22]. Mori et al. retrospectively analyzed 781 Japanese patients with RA in a hospital from the National Hospital Organization (NHO). ILD was detected on HRCT before or after the diagnosis of RA in 78 of the patients, and the mortality rates in patients with RA-ILD and RA without ILD were 2.1 and 1.16 times that of the Japanese general population, respectively. In a subanalysis of deceased patients, statistically significantly higher rates of lung cancer and respiratory failure as causes of death were found in patients with RA-ILD than RA without ILD [23]. The above-mentioned UIP and NSIP are found to be chronic conditions in RA-ILD, while organizing pneumonia and diffuse alveolar damage were identified as other subtypes that resulted in acute/subacute types [16].

In a survey for rheumatologists registered as ACR members conducted before publication of the 2023 guidelines, none of the respondents answered that they perform screening with HRCT [3]. However, in the 2023 guidelines for screening and monitoring of ILD by ACR/ACP, screening is conditionally recommended for patients with RDs who have an increased risk of developing ILD including RA. Among the listed screening methods, chest HRCT with and without PFT is given a conditional recommendation for screening, rather than history taking and physical examination or PFT [20]. In 2020, Japan was reported to have the largest number of CT units per million citizens out of all the countries in the Organization for Economic Co-operation and Development [24]; this might enable physicians in Japan to notice ILD at the early stages, and caution against frequent exposure to radiation is also needed. It is presumably particularly important for physicians in Japan who have easy access to CT units to consider both the benefits of the early detection of ILD and demerits of radiation exposure.

### 2.2. Predictive and Exacerbating Factors for RA-ILD

Older age, male sex, and smoking history are recognized as risk factors for RA-ILD [25]. In addition, the existence and severity of RA-ILD are closely related to the systemic disease activity of RA. High disease activity is known as a risk factor for ILD: in the above-mentioned study on patients in US hospitals with early RA, moderate-to-high disease activity was associated with ILD [17]. Rheumatoid factor (RF) and anti-cyclic citrullinated peptide antibody (ACPA) are items adopted in the 2010 ACR/EULAR classification criteria [26]. RF and ACPA measurements are useful not only for diagnosis of RA but also for predicting the effectiveness of bDMARDs such as tumor necrosis factor inhibitors (TNFis) and abatacept [27,28]. Positive RA and ACPA tests were reported to be associated with coexisting ILD: high-titer RF and ACPA are known as risk factors for RA-ILD [20]. However, a retrospective study by Li et al. reported no linear relationship between RA-ILD and titers of RF; rather, risk reduction was found in extremely high levels of RF, although confounding factors such as tight control of arthritis of high disease activity were presumedly involved [29].

Other than RF and ACPA, several autoantibodies specific to other RDs have been reported as predictive serum markers for ILD in patients with RA. Anti-aminoacyl-transfer ribonucleic acid synthetase (ARS) antibodies are myositis-specific antibodies (MSAs) found in polymyositis/dermatomyositis (DM); patients positive for anti-ARS antibodies have been known to present with antisynthetase syndrome, a series of symptoms including fever, Raynaud’s phenomenon, mechanic’s hand, myositis, and ILD [30]. Anti-Jo-1 antibodies, a class of anti-ARS antibodies, were adopted as a classification item in the 2017 ACR/EULAR criteria for idiopathic inflammatory myopathy [31], which is a concept including polymyositis, DM, inclusion body myositis, immune-mediated necrotizing myopathy, and so on [32]. MSA-including anti-ARS antibodies are not recognized as specific to RA, although anti-ARS antibodies were reported to be associated with ILD in patients with RA. Oka et al. measured serum levels of anti-ARS antibodies in patients with RA who visited NHO hospitals and underwent chest CT and found statistically significant higher titers of anti-ARS antibodies in patients with ILD compared to patients without chronic lung disease [33]. Anti-melanoma differentiation-associated gene 5 (MDA5) antibodies are another class of MSAs known for their high frequency of positiveness in clinically amyopathic DM, which often complicates rapidly progressive (RP)-ILD [34]. Oka et al. also studied anti-MDA5 antibodies in sera obtained from patients with RA (approved by the ethics committee of the NHO) and found that anti-MDA5 antibody titers showed an association with airway diseases, another form of chronic lung disease, but not significantly with ILD, which suggests that anti-MDA5 antibodies act differently in the lungs of patients with RA than in patients with DM [35]. However, there are several reports of Japanese cases developing DM with ILD positive for anti-MDA5 antibodies during treatment for RA with TNFis, as described later; physicians should pay attention to this possible complication of drug-induced ILD in the course of TNFi therapies [36,37]. Anti-Ro/SS-A antibodies were adopted as classification items in the 2016 ACR/EULAR criteria for Sjögren’s disease [38]. Anti-Ro/SS-A antibodies were reported to be positive in 3 to 15% of patients with RA; they affect the effectiveness of several classes of DMARD, including MTX and TNFis [39]. Ma et al. analyzed the influence of anti-Ro/SS-A antibodies in patients with RA, subdividing anti-Ro52 and anti-Ro60: double positiveness of anti-Ro52 and anti-Ro60 was found to be associated with high disease activity of RA, measured using disease activity score 28, a high erythrocyte sedimentation rate, and a higher risk of ILD [40]. Antinuclear antibody (ANA) was included as a required item in the 2019 ACR/EULAR classification criteria for systemic lupus erythematosus [41]. Nakano et al. analyzed 814 Japanese RA patients without other concomitant systemic RDs and found that positiveness of ANA with a nucleolar pattern was associated with lung complications, predominantly ILD [42].

Anti-neutrophil cytoplasmic antibodies (ANCAs) are clues used for diagnosis of ANCA-associated vasculitis (AAV), which is classified into granulomatous polyangiitis (GPA), microscopic polyangiitis (MPA), and eosinophilic granulomatous polyangiitis (EGPA). The lung is often affected, with ILD often present in AAV patients [43]. The ANCAs adopted in classification/diagnostic criteria for AAV are classified into cytoplasmic ANCA (cANCA) or perinuclear ANCA (pANCA) based on an indirect immunofluorescence pattern and proteinase 3-ANCA (PR3-ANCA) or myeloperoxidase-ANCA (MPO-ANCA) measured by chemiluminescent immunoassay [44]. cANCA, PR3-ANCA, pANCA, and MPO-ANCA are included in the 2022 ACR/EULAR classification criteria for GPA [45], MPA [46], and EGPA [47] (positive cANCA or PR3-ANCA are positively scored in GPA but negatively scored in MPA and EGPA, while positive pANCA or MPO-ANCA are positively scored in MPA but negatively scored in GPA). PR3-ANCA and MPO-ANCA are adopted in the diagnostic criteria by the Japanese Ministry of Health, Labour and Welfare for AAV, including GPA, MPA, and EGPA [48]. Huang et al. retrospectively analyzed positive rates of ANCAs in patients with RA-ILD and found that patients positive for ANCAs showed worse outcomes on ILD, such as decreased pulmonary function and more frequent acute exacerbation, compared to patients negative for ANCAs. In a subanalysis, patients positive for pANCA showed a tendency for shorter survival compared to patients negative for ANCAs [49]. Geographical differences have been reported in the prevalence of ANCAs: MPO-ANCA-positive AAV is relatively more common in Japan, where even GPA is often positive for MPO-ANCA [43]. Because the main target of pANCA is MPO, ANCA-positive RA-ILD might be a complication for which special attention is needed, especially for Japanese patients with RA. Anti-carbamylated protein antibodies are useful markers for diagnosing RA, even in cases negative for ACPA [50]. Castellanos-Moreira et al. analyzed 179 patients with RA and found significantly higher titers of anti-carbamylated proteins antibody in sera from patients with RA-ILD than in patients with RA without ILD [51]. Characteristics of autoantibodies reported as predictive serum markers for RA-ILD are summarized in Table 1.

In addition to autoantibodies, a couple of blood test items and environmental factors have been reported to have associations with RA-ILD. Uric acid (UA) acts as an antioxidant in the mucous membrane of airways. Wang et al. analyzed serum UA levels in patients with RA, and elevated serum UA levels were found in patients with RA-ILD with a usual interstitial pneumonia pattern [52]. Metalloproteinase-3, which is known as a serum marker associated with joint destruction in RA, was found to be higher in patients with RA-ILD than in those with RA without ILD in a retrospective study by Gou et al. In this study, the serum von Willebrand factor was also higher, which suggested a possibility that the pathophysiology of a disrupted pulmonary vascular barrier is present in RA-ILD [53]. Smoking is known as an exacerbating factor for RA-ILD [54]. Regarding genetic factors, the MUC5B promoter variant was reported to be associated with RA-ILD in recent studies [55,56]. Krebs von den Lungen-6, Surfactant Protein D, and metalloproteinase-7 were believed to be blood epithelial injury markers in RA-ILD [57]. Xu et al. developed a machine learning algorithm for predicting RA-ILD and reported that integrating Krebs von den Lungen-6 and other biomarkers would be useful for early diagnosis [58]. Other than the above-listed biomarkers, decreased forced vital capacity and diffusing capacity for carbon monoxide on PFT are associated with ILD progression [59,60].

## 3. Efficacy, Effectiveness, and Safety of Antirheumatic Drugs in Patients with ILD

### 3.1. Key Points of Treatment for RA-ILD

Whereas no specific recommendations on treatment for RA-ILD are currently available, several studies have suggested that the control of RA’s systemic disease activity is important even for RA-ILD [61]. Conflicting results such as the improvement or exacerbation of RA-ILD have been reported for every class of DMARD [62], but certain classes show better results. Therapeutic strategies are determined by the subtype of ILD, i.e., whether it is UIP or another subtype determines whether antifibrotic or anti-inflammatory drugs are indicated [63]. The existence of ILD highlights uncertainty regarding the effectiveness of DMARDs [64].

### 3.2. Glucocorticoid

In the current EULAR treatment recommendations for RA, it is advised that administration of glucocorticoid (GC) is tapered and stopped as early as possible [7]. In a nationwide study conducted in Taiwan, the average daily dose of prednisolone was identified as a risk factor of mortality in RA-ILD [65]. Considering the short-term benefit of GC, oral or intravenous administration is given conditional recommendation as a first-line treatment against ILD associated with RDs except systemic sclerosis, including RA, in the 2023 treatment guidelines by ACR/ACP. The short-term administration of GC, with the choice of administration route and dose depending on severity, is suggested for RA-ILD, while it is also mentioned that GC might not be used for RA-ILD with a UIP pattern [6]. In the guidelines of the ERS/EULAR, GC is listed as a treatment option for RA-ILD, whereas it is noted that long-term, high-dose administration should be avoided and GC-sparing treatments be considered [13]. In the 2020 diagnosis and treatment guide published by JRS/JCR, GC administration of different doses and routes is suggested according to the radiographic patterns and progression rates of RA-ILD [16]. The suggested doses of GC vary among guidelines because of the heterogeneous disease pictures of RA-ILD. The only consensus on the use of GC in treatment is early tapering and termination.

### 3.3. MTX

MTX is regarded as the anchor drug for treatment of RA due to its effectiveness and safety, even after the introduction of molecular target therapies into daily clinical practice [8]. In the latest recommendations by the EULAR [7] and in guidance from the Japan College of Rheumatology [9], it is recommended as the first-line DMARD on the condition that no contraindications are found in target patients. However, it is generally acknowledged that acute exacerbation of ILD associated with MTX, which needs to be diagnosed differently from MTX-induced pneumonitis, must be considered [66]. In the aforementioned survey conducted before publication of the 2023 guidelines for screening and monitoring of ILD by ACR/ACP [6], 72% of respondents answered they would avoid MTX for patients with ILD, and 62% of respondents would avoid for patients with abnormality on HRCT [3]. In the guidance from the Japan College of Rheumatology, chest radiography is recommended as a screening tool for ILD and lung infection before introduction of MTX, as well as for detecting adverse effects in the lung during administration of MTX [9].

Recently, several studies have reported beneficial effects of MTX on RA-ILD. In a study on patients with RA or psoriatic arthritis including five registries from Nordic countries, comedication of MTX was not found to be a risk factor of ILD associated with bDMARDs [10]. Izuka et al. retrospectively studied patients with RA-ILD in a Japanese population and found that a UIP pattern on HRCT was significantly associated with death, whereas MTX usage was associated with survival [11]. In a multicenter prospective study of RA-ILD conducted in Korea, in which the definition of progression was set as either 10% or more decrease in forced vital capacity, 15% or more decrease in diffusing capacity for carbon monoxide, or death of ILD or pneumonia, usage of MTX was not associated with progression [12]. There are other recent reports of positive results of MTX on RA-ILD [67,68].

MTX is the DMARD of first choice unless contraindicated, as mentioned above; however, the appropriateness of MTX for patients with RA is not established in cases with complicating ILD, which is a common extraarticular manifestation. Current guidelines give mixed results and opinions regarding the use of MTX on patients with RA with complication of ILD. The administration of MTX for patients with RA-ILD is no longer contraindicated uniformly but should be considered individually. Termination of MTX in case of exacerbation of lung symptoms is mentioned, and MTX is not included in treatment options against ILD associated with RA. Recommendations concerning MTX for patients with RA-ILD in guidelines from the ACR/ACP [6], ERS/EULAR [13], and JRS/JCR [16] are summarized in Table 2.

### 3.4. TAC

TAC, a class of calcineurin inhibitors, has been approved for the treatment of RA in some countries, including Japan [39]. In Japan, TAC is also approved for treatment against polymyositis/dermatomyoitis-associated ILD, the effectiveness of which was confirmed by interim analyses of postmarketing surveillance [69]. Ochi et al. reported a case of a Japanese patient with RA with complicated RP-ILD, in whom TAC was effective and enabled a reduced dose of GC [70]. In the 2020 diagnosis and treatment guidelines by the JRS/JCR, TAC is mentioned as a treatment for RA-ILD of NSIP or UIP [16], but it is not globally recognized as a promising therapeutic option. However, while TAC was associated with delayed progression of ILD in the aforementioned study of a South Korean population, this was not statistically significant [12]. There is a case report of drug-induced acute exacerbation of RA-ILD associated with TAC, which suggests a need for caution against drug-induced exacerbation, even during administration of TAC [71].

### 3.5. TNFis

TNFis are a class of bDMARDs prescribed as frequently as MTX [3]. Whereas even TNFi monotherapy is effective for RA, several clinical trials have revealed that therapeutic effects of TNFis would be maximized when administered in combination with MTX [8].

The influence of TNFis on RA-ILD was analyzed based on records from the British Society for Rheumatology in the 2000s, and TNFis were not found to increase mortality, despite the rate of deaths due to ILD being higher in patients treated with TNFis compared to patients treated with traditional DMARDs [72]. Several classes of TNFi have been suspected of causing acute exacerbation of RA-ILD. Cases of acute exacerbation of RA-ILD induced by adalimumab in combination with MTX have been reported [73,74]. In addition to combination therapy of TNFis and MTX, there are case reports of the acute exacerbation of RA-ILD after the introduction of etanercept (ETN) after termination of MTX [75] and ETN monotherapy [76]. There is a case report of RA-ILD induced by certolizumab pegol, a class of TNFis composed of polyethylene glycolylated Fab fragments [77]. There are currently no reports of RA-ILD induced by ozoralizumab, a newly introduced TNFi [78]. In the aforementioned survey of ACR members, 24% of respondents answered that they would avoid TNFis for patients with ILD and 31% of respondents would avoid them for patients with abnormality on HRCT [3].

There are several case reports from Japan describing TNFi-associated acute exacerbation of ILD using anti-MDA5 antibodies. Matsumoto et al. reported a Japanese case of acute exacerbation of ILD positive for anti-MDA5 antibodies during treatment of ADA in combination with MTX and salazosulfapyridine [36]. Okazaki et al. reported a Japanese case of developing DM positive for anti-MDA5 antibodies during treatment of ETN biosimilar [37]. These case reports suggested that anti-MDA5 antibodies might be a latent risk factor for RA-ILD, which has not been ascertained by clinical studies with large patient numbers. A geographical difference in complication rates of ILD in patients with DM positive for anti-MDA5 antibodies has been reported: in a comparison between patients with DM positive for anti-MDA5 antibodies in a Brazilian population and a Japanese population, the complication rate of ILD was lower in the Brazilian population, especially the complication rate of RP-ILD [79]. Studies on the effectiveness and safety of TNFis for RA-ILD are limited and insufficient to draw conclusions. Further studies are warranted to clarify this matter.

### 3.6. Abatacept

Fernández-Díaz et al. reported results of a retrospective, multicenter study analyzing the efficacy and safety of abatacept (ABT) in Caucasian patients with RA-ILD separated into three groups: ABT with MTX; ABT monotherapy; and ABT with other cDMARDs. Scores were comparable among the groups for each outcome—such as the disease activity score 28 erythrocyte sedimentation rate—but were better in the combination groups, i.e., ABT with MTX or ABT with other csDMARDs [80]. Dieudé et al. reported the results of an analysis combining ten clinical trials on the effects of ABT in combination with MTX or MTX with placebo for RA, in which the ABT in combination with MTX group showed a lower rate of ILD than MTX with placebo [81]. Shoda et al. conducted a retrospective analysis of patients with RA-ILD who were treated in a Japanese university hospital and found that ABT was effective for improvement in ground glass opacity scores, even in fibrosis scores of UIP on HRCT findings [82].

Conversely, there are other studies of the progression of RA-ILD during administration of ABT, in which the effects of concomitant use of MTX are not coherent. Serrano-Combarro et al. conducted a multicenter study of 40 hospitals in Spain on patients with RA-ILD treated with ABT, and progression of ILD was found in around one quarter of the patients. In the subanalyses, prior MTX administration was found to be a protective factor, while administration of TNFis was found to be a risk factor of progression [83]. However, in a study conducted in a Japanese university hospital, 8.4% of patients who were treated with ABT showed exacerbation of RA-ILD, and concomitant administration of MTX was identified as a risk factor of exacerbation. The authors suggested that withdrawal of MTX should be considered when remission of RA was achieved with ABT [84]. To date, studies on RA-ILD and ABT have primarily consisted of observational studies with different populations and designs and should thus be interpreted with caution by physicians.

### 3.7. Anti-Interleukin 6 Inhibitors

Tocilizumab (TCZ) is an interleukin-6 receptor inhibitor (IL-6i). TCZ has been reported as effective even without concomitant MTX in a multicenter prospective study in Japan [85], which suggests that TCZ is a plausible option for patients for whom MTX is not appropriate due to ILD. It was reported that TCZ might have a stabilizing effect against RA-ILD. Manfredi et al. analyzed patients with RA-ILD who received TCZ treatment with or without MTX retrospectively, most of whom showed stable HRCT findings during follow-up periods [86].

However, there are reports of drug-induced exacerbation of RA-ILD during administration of TCZ. Kawashiri et al. reported the first case of acute exacerbation of RA-ILD after introduction of TCZ as an alternative to ETN [87]. Akiyama et al. analyzed patients with RA-ILD who were treated with TCZ and found that a high clinical disease activity index of RA at 24 weeks of TCZ administration was significantly associated with acute exacerbation of ILD, suggesting the activity of arthritis has an association with worsening ILD in this population [88].

Sarilumab (SAR) is another IL-6i that has been reported as having potential beneficial effects against RA-ILD. Suzuki et al. analyzed patients with RA who received SAR treatment; in most of them, ILD was stable during SAR administration, and two showed improvements in ILD as well as arthritis. Exacerbation was observed in one patient [89]. To date, the studies on RA-ILD and IL-6is primarily consist of observational studies, and suppressive effects on ILD in patients with RA have yet to be established.

### 3.8. JAKis

JAKis are considered as effective for RA even without concomitant MTX, whereas combination therapy would produce additional effects. Kurushima et al. reported that baricitinib treatment in combination with MTX significantly suppressed epithelial–mesenchymal transition, reflecting pulmonary fibrosis in vitro [90]. In a clinical study, Suzuki et al. retrospectively analyzed treatment courses in patients with RA-ILD treated with five classes of JAKis (tofacitinib, baricitinib, peficitinib, upadacitinib, and filgotinib) in a Japanese population and found comparable retention rates of ILD and arthritis between the patients treated with JAKis with MTX and those without MTX [91].

Recently, tofacitinib has been reported to show beneficial effects on RA-ILD when administered with iguratimod [92,93]. There are reports suggesting beneficial effects of filgotinib without concomitant use of MTX on RA-ILD. Sunaga et al. reported a case of RA-ILD for which filgotinib was effective when switched from abatacept [94]. Sato et al. reported a patient with RA preceded by ILD, for whom filgotinib as an addition to salazosulfapyridine decreased disease activity of RA and Krebs von den Lungen-6 levels [95]. Nishii et al. reported a case of progressive RA-ILD refractory to incremental prednisolone and a switch from MTX to TAC, ABT, and intravenous cyclophosphamide, in which upadacitinib effectively improved ILD as well as disease activity of RA [96]. However, the therapeutic effectiveness of JAKis in RA-ILD has yet to be validated in clinical trials with large patient numbers. Furthermore, the risk of adverse effects of JAKis such as malignancy and major adverse cardiovascular events, which were revealed in ORAL Surveillance of tofacitinib [97], should be noted for patients with RA-ILD.

### 3.9. Rituximab

Rituximab is classified as a bDMARD for RA treatment. Although RTX has not been approved for RA in Japan, several studies’ results suggest that RTX would be an appropriate option for RA-ILD [98].

### 3.10. Antifibrotic Agents

The number of studies showing the effectiveness of antifibrotic agents has recently been increasing. Solomon et al. reported results of phase 2 trials suggesting that pirfenidone effectively slowed the decline in forced vital capacity and was safe in RA-ILD [99]. Nitedanib, another type of antifibrotic agent, has also been suggested for treating ILD associated with rheumatic disease including RA [100], with the results of relevant studies being introduced in the ERS/EULAR guidelines [13].

### 3.11. Leflunomide

Leflunomide has been utilized as a type of DMARD; however, it is assumed to be associated with drug-induced pneumonitis and ILD exacerbation. It has been stated in JRS/JCR guidelines that leflunomide should not be used for RA-UIP [16].

## 4. Conclusions

ILD is a complication that affects the overall survival of patients with RA and the choice of DMARDs for treatment. Attending physicians should be cautious of this when they encounter patients with RA, and HRCT should be used to detect ILD as necessary. Although MTX, the anchor drug for treatment for RA, has been considered inappropriate for patients with RA-ILD, there are recent reports of the beneficial effects of MTX on RA-ILD. Conversely, studies have reported that several kinds of bDMARDs, which were considered tolerable for patients with RA-ILD, might worsen ILD. While ABT, TCZ, and RTX seem relatively preferable according to previous studies [101], further studies are warranted to confirm this. It is possible that these contradictory results regarding one class of DMARDs are attributable to confounding factors such as study design and selected populations. The above-mentioned reports on anti-MDA5 antibodies in RA-ILD suggest that there may be underlying ethnic factors influencing RA-ILD. Rheumatologists should be aware of emergence or changes in activity of ILD and arthritis during treatment with any kind of DMARD.

## Figures and Tables

**Table 1:** Characteristics of autoantibodies reported as predictive serum markers for RA-ILD.

Autoantibodies	Characteristics	References
Diagnostic markers of RA as well as widely known risk factors of RA-ILD		
RF	Adopted as classification items in 2010 ACR/EULAR classification criteria for RA High-titer RF is known as a risk factor for RA-ILD	[20,26]
ACPA	Adopted as classification items in 2010 ACR/EULAR classification criteria for RA High-titer ACPA is known as a risk factor for RA-ILD	[20,26]
Potentially contributing autoantibodies in RA-ILD		
Anti-ARS antibodies	Known for autoantibodies of PM/DM, anti-ARS syndrome Reported to be associated with RA-ILD in a Japanese population	[30,33]
	Anti-Jo-1 antibodies adopted in 2017 ACR/EULAR criteria for IIM	[31]
Anti-MDA5 antibodies	Known for autoantibodies of RP-ILD associated with DM Several reports of Japanese cases developing DM with ILD positive for anti-MDA5 antibodies during treatment for RA with TNFis	[34,36,37]
Anti-Ro/SS-A antibodies	Adopted as classification items in 2016 ACR/EULAR classification criteria for SjD Double positiveness of anti-Ro52 and anti-Ro60 was found to be associated with higher risk of ILD in a Chinese population	[38,40]
ANA	Adopted as required item in 2019 ACR/EULAR classification criteria for SLE Positiveness of ANA with a nucleolar pattern was associated with lung complications, predominantly ILD, in a Japanese population	[41,42]
ANCA	Adopted as classification items in 2022 ACR/EULAR classification criteria for	[45,46,47,48,49]
	ANCA-associated vasculitis (GPA, MPA and EGPA) Positivity for ANCAs was found to be associated with worse outcomes on ILD, such as decreased pulmonary function and more frequent acute exacerbation in a Chinese population of RA	

ACPA, anti-cyclic citrullinated peptide antibody; ACR, American College of Rheumatology; ANA, antinuclear antibody; ANCA, anti-neutrophil cytoplasmic antibodies; ARS, aminoacyl-transfer ribonucleic acid synthetase; DM, dermatomyositis; EGPA, eosinophilic granulomatous polyangiitis; EULAR, European Alliance of Associations for Rheumatology; GPA, granulomatous polyangiitis; IIM, idiopathic inflammatory myopathy; ILD, interstitial lung disease; MDA5, melanoma differentiation-associated gene 5; MPA, microscopic polyangiitis; PM, polymyositis; RA, rheumatoid arthritis; RF, rheumatoid factor; RP, rapidly progressive; SjD, Sjögren’s disease; SLE, systemic lupus erythematosus; TNFis, tumor necrosis factor inhibitors.

**Table 2:** Recommendations concerning MTX for patients with RA-ILD in guidelines from ACR/ACP, ERS/EULAR, and JRS/JCR.

ACR/ACP [6]	(Described as for ILD associated with systemic autoimmune rheumatic diseases) MTX can be continued in case of prescription for extrapulmonary symptoms but should be terminated when MTX pneumonitis is suspected. Evidence of beneficial effects of MTX for ILD is not established. MTX is not included in treatment options for RA-ILD.
ERS/EULAR [13]	Listing both results regarding MTX for RA-ILD: MTX is not included in treatment options for RA-ILD.
JRS/JCR [16]	MTX is listed as one of the risk factors for acute exacerbation of RA-ILD, and termination of suspected drugs causing exacerbation, both in mild and severe cases, is mentioned. MTX is not included in treatment options for RA-ILD.

ACP, American College of Chest Physicians; ACR, American college of Rheumatology; ERS, European Respiratory Society; EULAR, European Alliance of Associations for Rheumatology; ILD, interstitial lung disease; JCR, Japan College of Rheumatology; JRS, Japanese Respiratory Society; RA, rheumatoid arthritis.

## Data Availability

No new data were created or analyzed in this study.
